# Timeliness of Receipt of Early Childhood Vaccinations Among Children of Immigrants — Minnesota, 2016

**DOI:** 10.15585/mmwr.mm6642a1

**Published:** 2017-10-27

**Authors:** Maureen Leeds, Miriam Halstead Muscoplat

**Affiliations:** 1 Minnesota Department of Health, Infectious Disease Epidemiology, Prevention and Control.

Receiving recommended childhood vaccinations on schedule is the best way to prevent the occurrence and spread of vaccine-preventable diseases (*1*). Vaccination coverage among children aged 19–35 months in the United States exceeds 90% for most recommended vaccines in the early childhood series (*2*); however, previous studies have found that few children receive all recommended vaccine doses on time (*3*). The Minnesota Department of Health (MDH), using information from the Minnesota Immunization Information Connection (MIIC) and the MDH Office of Vital Records, examined early childhood immunization rates and found that children with at least one foreign-born parent were less likely to be up-to-date on recommended immunizations at ages 2, 6, 18, and 36 months than were children with two U.S.-born parents. Vaccination coverage at age 36 months varied by mother’s region of origin, ranging from 77.5% among children born to mothers from Central and South America and the Caribbean to 44.2% among children born to mothers from Somalia. Low vaccination coverage in these communities puts susceptible children and adults at risk for outbreaks of vaccine-preventable diseases, as evidenced by the recent measles outbreak in Minnesota (*4*). Increased outreach to immigrant, migrant, and refugee populations and other populations with low up-to-date vaccination rates might improve timely vaccination in these communities.

A retrospective cohort study was conducted using existing birth certificate data from the Office of Vital Records and vaccination records from MIIC. The Office of Vital Records maintains electronic records for all births occurring in Minnesota. MIIC is a statewide immunization information system that includes vaccination records for children and adults residing in Minnesota. Most health care providers in Minnesota routinely submit data to MIIC; 92% of Minnesota children aged 24–35 months have at least two noninfluenza vaccination records in the system.*

Birth records for children born in Minnesota during 2011–2012 were obtained from the Office of Vital Records and matched to immunization records by MIIC personnel in November 2016 using birth certificate numbers. All records were for children aged ≥36 months. The information of primary interest was foreign birth of one or both parents, stratified by mother’s region of origin. This information was ascertained from birth records collected by the Office of Vital Records shortly after birth. The primary outcome of interest was the receipt of recommended vaccines at ages 2, 6, 18, and 36 months,^†^ following the current recommendations of the Advisory Committee on Immunization Practices.^§^^,^^¶^ Parental demographic characteristics were obtained from birth records maintained by the Office of Vital Records, including race, age, education, country of birth, maternal state of residence, and whether the mother participated in the Women, Infants, and Children (WIC) program during pregnancy. The study protocol was reviewed by the University of Minnesota Institutional Review Board, and deemed exempt from requirement for human subjects research approval.

Children were categorized into the following regional groups, based on their mother’s birth country: United States, Asia, Eastern Europe, Western Europe and Canada, Africa (excluding Somalia), Central and South America and the Caribbean, and Oceania/Other. Somalia and Mexico, the two largest groups of foreign-born mothers of children in the sample, were considered separately. All analyses were performed using statistical software. Statistical significance was set at p<0.05, using two-sided tests. Multivariate logistic regression models were adjusted for the following variables: maternal age, race, and educational attainment. These were then used to estimate unadjusted and adjusted odds ratios for up-to-date vaccinations recommended at ages 2, 6, 18, and 36 months, comparing children with at least one foreign-born parent with children with two U.S.-born parents.

Vaccination records and parental characteristic information were obtained for 135,389 children. Removed from the merged data set were 36,998 records with missing or unknown data on parental countries of birth, maternal state of residence, WIC participation status during pregnancy, parental age, education, or race; children whose status was “not living” or “unknown” at time of birth record filing (150 children); and children born before 24 weeks’ or after 42 weeks’ gestation or whose gestational age was unknown (356), leaving a final sample of 97,885 (72.3%). Overall, 22% of children had at least one foreign-born parent, 30% of mothers participated in WIC during pregnancy, 80% of mothers were aged 20–34 years, nearly 80% were white, and 75% had attended at least some college (Table 1).

**TABLE 1:** Percentage of children born during 2011–2012 who were up-to-date with recommended vaccinations at ages 2, 6, 18, and 36 months, by selected maternal characteristics* — Minnesota, 2016

Characteristic	Total	Age vaccinations were up-to date			
		2 mos	6 mos	18 mos	36 mos
	No. (%)	No. (%)	No. (%)	No. (%)	No. (%)
**At least one parent foreign-born**	**21,579 (22.1)**	**13,768 (63.8)**	**9,973 (46.2)**	**7,239 (33.6)**	**14,112 (65.4)**
**Both parents U.S.-born**	**76,306 (77.9)**	**48,767 (63.9)**	**39,219 (51.4)**	**31,594 (41.4)**	**54,400 (71.3)**
**Mother participated in WIC program during pregnancy**					
Yes	29,495 (30.1)	20,594 (69.8)	14,514 (49.2)	9,753 (33.1)	20,735 (70.3)
No	68,390 (69.9)	41,941 (61.3)	34,678 (50.7)	29,080 (42.5)	47,777 (69.9)
**Mother’s age (yrs)**					
≤19	2,943 (3.0)	2,117 (71.9)	1,441 (49.0)	908 (30.9)	2,134 (72.5)
20–34	79,494 (81.2)	51,551 (64.9)	40,665 (51.2)	32,068 (40.3)	55,950 (70.4)
≥35	15,448 (15.8)	8,867 (57.4)	7,086 (45.9)	5,857 (37.9)	10,428 (67.5)
**Maternal race**					
White	77,203 (78.9)	49,052 (63.5)	39,932 (51.7)	32,210 (41.7)	54,743 (70.9)
Black	6,928 (7.1)	4,477 (64.6)	2,837 (41.0)	1,735 (25.0)	4,241 (61.2)
Other	13,754 (14.0)	9,006 (65.5)	6,423 (46.7)	4,888 (35.5)	9,528 (69.3)
**Mother's education attainment**					
≤12th grade, no diploma	8,179 (8.4)	5,505 (67.3)	3,600 (44.0)	2,319 (28.4)	5,403 (66.1)
High school diploma or GED	14,447 (14.8)	9,711 (67.2)	6,870 (47.6)	4,615 (31.9)	9,800 (67.8)
Associate degree/College credit	32,160 (32.9)	21,355 (66.4)	16,608 (51.6)	12,156 (37.8)	22,722 (70.7)
Bachelor’s degree or higher	43,099 (44.0)	25,964 (60.2)	22,114 (51.3)	19,743 (45.8)	30,587 (71.0)
**Total**	**97,885 (100)**	**62,535 (63.9)**	**49,192 (50.3)**	**38,833 (39.7)**	**68,512 (70.0)**

**Abbreviations:** GED = General Educational Development; WIC = Special Supplemental Nutrition Program for Women, Infants, and Children.

* Information from the Minnesota Department of Health Office of Vital Records.

Birth of one or both parents outside the United States was significantly associated with a child’s not being up-to-date on vaccinations at ages 2, 6, and 18 months, and not being caught up by age 36 months (Figure). There were differences in children’s up-to-date status by mother’s region of birth. The percentage of children up-to-date at all ages was higher among those whose mothers were born in Central and South America and the Caribbean, Mexico, and Africa (excluding Somalia) than the percentage among children of U.S.-born mothers. In every maternal regional category the percentage of children up-to-date declined from age 2 months to age 6 months and from age 6 months to age 18 months; however, except for children of Somali-born mothers, the percentage of children up-to-date at age 36 months was as high or higher than that at age 2 months. The lowest percentage of children up-to-date at ages 2, 6, and 18 months were those with mothers born in Eastern Europe; just over half of children whose mothers were born in Eastern Europe were up to date at age 36 months. Fewer than 10% of children whose mothers were born in Somalia were up-to-date at 18 months, although by 36 months, 44.2% had caught up.

**FIGURE:**
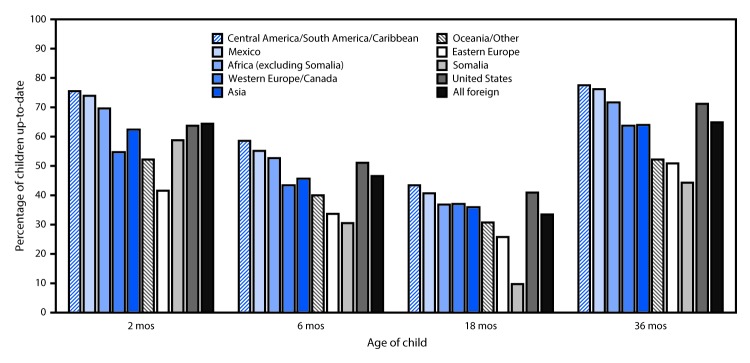
Percentage of children born during 2011–2012 who were up-to-date on recommended vaccinations at ages 2, 6, 18, and 36 months, by mother’s birth region — Minnesota, 2016* * Total number of children born in Minnesota during 2011–2012, by mother’s birth region: United States, 80,664; all foreign, 17,221; Africa (excluding Somalia), 2,521; Asia, 6,463; Central and South America/Caribbean, 1,445; Eastern Europe, 802; Mexico, 2,712; Oceana/Other, 65; Somalia, 2,321; Western Europe and Canada, 892.

Overall, children with at least one foreign-born parent were 25% less likely to be current on their vaccinations at 36 months than were children born to two U.S.-born parents, after adjusting for maternal race, age, and educational attainment (Table 2). Participation in WIC during pregnancy was significantly associated with being up-to-date at 2, 6, and 36 months. Children born to mothers from Africa (excluding Somalia), Central and South America and the Caribbean, and Mexico were significantly more likely to be up-to-date at ages 2, 6, 18, and 36 months compared with children with U.S.-born mothers. Children born to mothers from all other regions (Western Europe and Canada, Eastern Europe, Asia, and Somalia) were significantly less likely to be up-to-date at all ages than were children with U.S.-born mothers. Children with mothers from Somalia and Eastern Europe were least likely to be up-to-date at all ages.

**TABLE 2:** Unadjusted and adjusted* odds ratios (ORs) for up-to-date recommended vaccination status at ages 2, 6, 18, and 36 months among children born during 2011–2012, comparing children with at least one foreign-born parent with children with two U.S.-born parents — Minnesota, 2016

Characteristic	Age vaccinations were up-to date			
	2 mos	6 mos	18 mos	36 mos
	OR (95% CI)	OR (95% CI)	OR (95% CI)	OR (95% CI)
**Foreign born parent(s)**				
Unadjusted	0.99 (0.96–1.03)^†^	0.81 (0.79–0.84)	0.71 (0.69–0.74)	0.76 (0.74–0.79)
Adjusted	0.93 (0.90–0.96)	0.87 (0.84–0.90)	0.82 (0.79–0.85)	0.75 (0.72–0.78)
**WIC during pregnancy**				
Unadjusted	1.46 (1.42–1.50)	0.94 (0.92–0.97)	0.67 (0.65–0.69)	1.02 (0.99–1.05)^†^
Adjusted	1.37 (1.32–1.42)	1.04 (1.01–1.08)	0.87 (0.84–0.90)	1.15 (1.11–1.19)
**Foreign-born mothers birth region (adjusted OR [95% CI])**				
Central and South America/Caribbean	1.65 (1.45–1.87)	1.70 (1.53–1.90)	1.71 (1.53–1.91)	1.61 (1.41–1.83)
Mexico	1.45 (1.31–1.60)	1.63 (1.49–1.78)	1.84 (1.68–2.02)	1.58 (1.42–1.75)
Africa (excluding Somalia)	1.27 (1.17–1.40)	1.61 (1.07–1.26)	1.03 (0.95–1.12)	1.12 (1.02–1.22)
Western Europe and Canada	0.74 (0.65–0.85)	0.75 (0.66–0.86)	0.80 (0.70–0.92)	0.72 (0.63–0.83)
Asia	0.97 (0.91–1.03)^†^	0.93 (0.88–0.99)	0.94 (0.88–0.99)	0.74 (0.70–0.79)
Eastern Europe	0.41 (0.36–0.47)	0.49 (0.42–0.57)	0.49 (0.42–0.57)	0.43 (0.37–0.49)
Somalia	0.70 (0.64–0.76)	0.49 (0.45–0.54)	0.25 (0.21–0.28)	0.38 (0.25–0.41)

**Abbreviations:** CI = confidence interval; WIC = Special Supplemental Nutrition Program for Women, Infants, and Children.

* Adjusted for maternal race, age, and education.

^†^ OR is not statistically significant (p≥0.05).

## Discussion

This study found wide variation in up-to-date vaccination status at different ages among Minnesota children with U.S.-born parents and those with at least one foreign-born parent. Up-to-date status varied by the mother’s country of origin, with children of mothers born in Eastern Europe, Western Europe and Canada, and Somalia being less likely than children with U.S.-born mothers to be up-to-date at all ages, and those with mothers born in African countries (excluding Somalia), Central and South America and the Caribbean, and Mexico being more likely than children with U.S.-born mothers to be up-to-date at all ages. Inadequate parental understanding of vaccination and weaker public health education programs in some regions might account for some of these findings, as well as economic and social factors influencing emigration, including fleeing war, religious persecution, or poverty (*5*). Somali parents in Minnesota have been reported to be more likely than non-Somali parents to have concerns about the safety of measles-mumps-rubella (MMR) vaccine, which has led to a decline in coverage with MMR and possibly other childhood vaccines (*6*). From April to August 2017, Minnesota experienced a measles outbreak, ending with 79 confirmed cases, including 65 in children of Somali descent (*4*).

The findings in this report are subject to at least three limitations. First, health care provider participation in MIIC is voluntary, and MIIC might not account for children who receive immunizations in bordering states (excluding Wisconsin and North Dakota, which do exchange immunization data). Second, because of the nature of the data used, information on the health status of children in the study after birth was not available; therefore, it was not possible to determine whether any child had a medical contraindication to vaccination. Finally, the information gathered by the Office of Vital Records on parental countries of origin is self-reported and did not include information on when the parent arrived in the United States.

Participation in WIC was associated with an increased likelihood of up-to-date vaccination status, and engaging eligible foreign-born families in programs such as WIC might provide an opportunity to increase on-time vaccination (*7*). Focus groups, meetings, and conversations with the Somali community have been employed in an effort to understand the underlying reasons for low vaccination rates; similar work could be done with the Eastern European immigrant community and other populations with low immunization coverage or late vaccination. Possible strategies include outreach to community leaders, parents, interpreters, and spiritual leaders to provide information on vaccines and vaccine preventable diseases. Encouraging medical providers to use interpreters, take time to build trust, and assess vaccination status at every visit might improve vaccination coverage in these populations (*8*).

**Summary**

**What is already known about this topic?**

Receiving the recommended childhood vaccinations on schedule is the best way to prevent vaccine-preventable diseases. Vaccination coverage in the United States for children aged 19–35 months exceeds 90% for most recommended childhood vaccines. Previous studies have found that few children receive all their vaccinations on time; however, few studies have examined whether a mother’s country of birth affects her child’s up-to-date vaccination status at various ages.

**What is added by this report?**

Fewer than half of children born in Minnesota in 2011–2012 were up-to-date on their immunizations at 18 months, and only 70% were caught up by 36 months. Up-to-date vaccination status was lower among children with at least one foreign-born parent compared with that of children with two U.S.-born parents, and rates varied by mother’s country of origin. Children with mothers born in Somalia and Eastern Europe had the lowest rates of up-to-date vaccination.

**What are the implications for public health practice?**

Refugees and immigrants to the United States from certain regions might have greater difficulties getting their children vaccinated in a timely manner, compared with U.S.-born parents and parents from some other countries. Increased outreach to Eastern European and Somali immigrant, migrant, and refugee populations might benefit children in these communities by improving on-time receipt of recommended vaccinations.

## Footnotes

*
http://www.health.state.mn.us/miic .

† Age in months automatically calculated by the Minnesota Immunization Information Connection. For example, the 2 months category includes 2 months and zero days through to the day before the child reaches 3 months of age.

§
https://www.cdc.gov/vaccines/schedules/downloads/child/0-18yrs-child-combined-schedule.pdf .

¶ Up-to-date includes receipt of age-appropriate doses of hepatitis B vaccine, diphtheria and tetanus toxoids and acellular pertussis vaccine (DTaP), *Haemophilus influenzae* type b (Hib) vaccine, pneumococcal conjugate vaccine, polio vaccine, measles-mumps-rubella (MMR) vaccine, and varicella vaccine; and takes into account the number of doses recommended by the Advisory Committee on Immunization Practices as well as the minimum age and interval requirements.
